# Extraction of a stent–stone complex with a 9-French cholangioscope during electrohydraulic lithotripsy

**DOI:** 10.1055/a-2489-8141

**Published:** 2024-12-10

**Authors:** Koichi Soga, Yuki Soma, Kazuma Sakakibara, Takeshi Fujiwara, Fuki Hayakawa, Ikuhiro Kobori, Masaya Tamano

**Affiliations:** 1 26263 Department of Gastroenterology, Dokkyo Medical University Saitama Medical Center, Koshigaya, Japan

A stent-stone complex (SSC), a rare complication of long-term biliary plastic stent (PS) placement (mainly caused by a forgotten, retained PS), complicates endoscopic SSC removal 1 . We report a case of successful endoscopic removal of an SSC using peroral cholangioscopy (POCS)-guided electrohydraulic lithotripsy (EHL).

An 89-year-old woman was referred to our hospital for the treatment of cholangitis and common bile duct (CBD) stones with an SSC. The large number of CBD stones made their removal challenging. Three years before presentation, two PSs were inserted into the CBD at a previous hospital. Abdominal computed tomography revealed a large CBD stone that resembled a lollipop and formed a complex at the tip of the two PSs 2 . The PSs broke off at the duodenal lumen during our removal attempt using an endoscopic snare ( Fig. 1 ). Therefore, we performed POCS-guided EHL with a 9-Fr cholangioscope (eyeMAX; Micro-Tech, Nanjing, China) to extract the SSC. During POCS, the SSC was observed in the CBD. The stones were stabilized by the PSs, enabling efficient shock wave delivery. The CBD stones were crushed during the EHL procedures; the crushed stones and PSs were removed using an endoscopic lithotripsy device ( Fig. 2 , Video 1 ). The patient’s clinical course was uneventful.

**Fig. 1:**
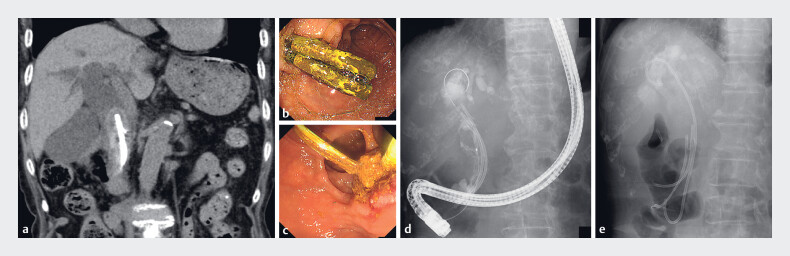
Images of the stent-stone complex (SSC) with choledocholithiasis in the common bile duct (CBD) and the first endoscopic retrograde cholangiopancreatography (ERCP) procedure at our hospital. **a** Abdominal computed tomography reveals a large CBD stone that resembles a lollipop and forms a complex at the tip of two plastic stents (PSs) with a 21.4-mm dilated CBD around the stent. **b–e** The first ERCP procedure at our hospital. **b** Endoscopy images confirming the presence of two PSs that were inserted into the CBD 3 years previously at another hospital. **c** An attempt to remove the PSs using an endoscopic snare was unsuccessful. The PSs broke off at the duodenal lumen. **d** ERCP images showing a large translucent filling defect around the stent in the CBD. **e** We decided to abandon our attempt to remove the primary CBD stones and PSs; instead, we performed electrohydraulic lithotripsy.

**Fig. 2:**
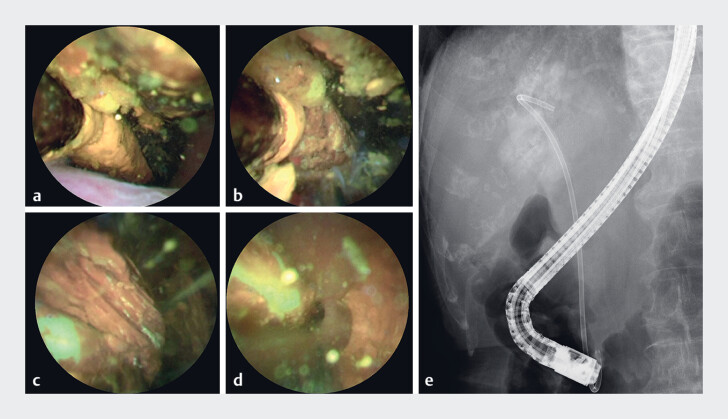
Peroral cholangioscopy (POCS)-guided electrohydraulic lithotripsy (EHL) for the removal of the SSC. **a–d** Images obtained during POCS. **a** The SSC, which resembles a lollipop, is observed in the CBD. **b** The stones are stabilized with PSs, enabling the efficient delivery of shock waves. **c** An EHL device carefully broke the stones stuck in the stent. **d** After the stones were crushed, the mobility of the SSC improved and the PSs were free and floating in the CBD. **e** The stones were crushed during the EHL procedures; the crushed stones and PSs were removed using an endoscopic lithotripsy device. Cholangiography confirmed the absence of residual stones.

Extraction of a stent-stone complex with a 9-Fr cholangioscope during electrohydraulic lithotripsy.Video 1

The 9-Fr cholangioscope, which was thinner than those used for previous cases, used during POCS enabled easier access to the CBD and SSC and straightforward performance of endoscopic procedures 3 . We believe that direct visualization during POCS-guided EHL is extremely useful because it enables the evaluation and removal of the SSC. A long stenting period (>300 days) and large CBD diameter during stent placement are independent risk factors for SSC formation 4 . The possibility of an SSC should be considered for cases involving a dilated CBD and prolonged PS placement.

Endoscopy_UCTN_Code_TTT_1AR_2AH

## Notes

Endoscopy E-Videos https://eref.thieme.de/e-videos
*E-Videos* is an open access online section of the journal *Endoscopy* , reporting on interesting cases and new techniques in gastroenterological endoscopy. All papers include a high-quality video and are published with a Creative Commons CC-BY license. Endoscopy E-Videos qualify for HINARI discounts and waivers and eligibility is automatically checked during the submission process. We grant 100% waivers to articles whose corresponding authors are based in Group A countries and 50% waivers to those who are based in Group B countries as classified by Research4Life (see: https://www.research4life.org/access/eligibility/ ). This section has its own submission website at https://mc.manuscriptcentral.com/e-videos .
